# IL-6 and the dysregulation of immune, bone, muscle, and metabolic homeostasis during spaceflight

**DOI:** 10.1038/s41526-018-0057-9

**Published:** 2018-12-04

**Authors:** John Kelly Smith

**Affiliations:** Departments of Academic Affairs and Biomedical Sciences, James H. Quillen College of Medicine, East Tennessee State University, Johnson City, TN USA

## Abstract

We have previously reported that exercise-related secretion of IL-6 by peripheral blood mononuclear cells is proportionate to body weight, suggesting that IL-6 is gravisensitive and that suboptimal production of this key cytokine may contribute to homeostatic dysregulations that occur during spaceflight. This review details what is known about the role of this key cytokine in innate and adaptive immunity, hematopoiesis, and in bone, muscle and metabolic homeostasis on Earth and in the microgravity of space and suggests an experimental approach to confirm or disavow the role of IL-6 in space-related dysregulations.

## Introduction

In 2016, NASA’s Human Research Program solicited research to help address health issues associated with spaceflight, including immune dysregulation and the risk of early onset osteoporosis and substandard performance due to reductions in muscle mass, endurance, and strength.^1^

Currently, high intensity resistance and aerobic exercises are recognized as being the most effective countermeasures available to astronauts to mitigate the adverse effects of microgravity on bone and skeletal muscle.^2^ However, despite the implementation of high impact exercise training programs, unacceptable bone loss and muscle atrophy continue to occur during both short-term and extended spaceflight missions.^3^

**Fig. 1:**
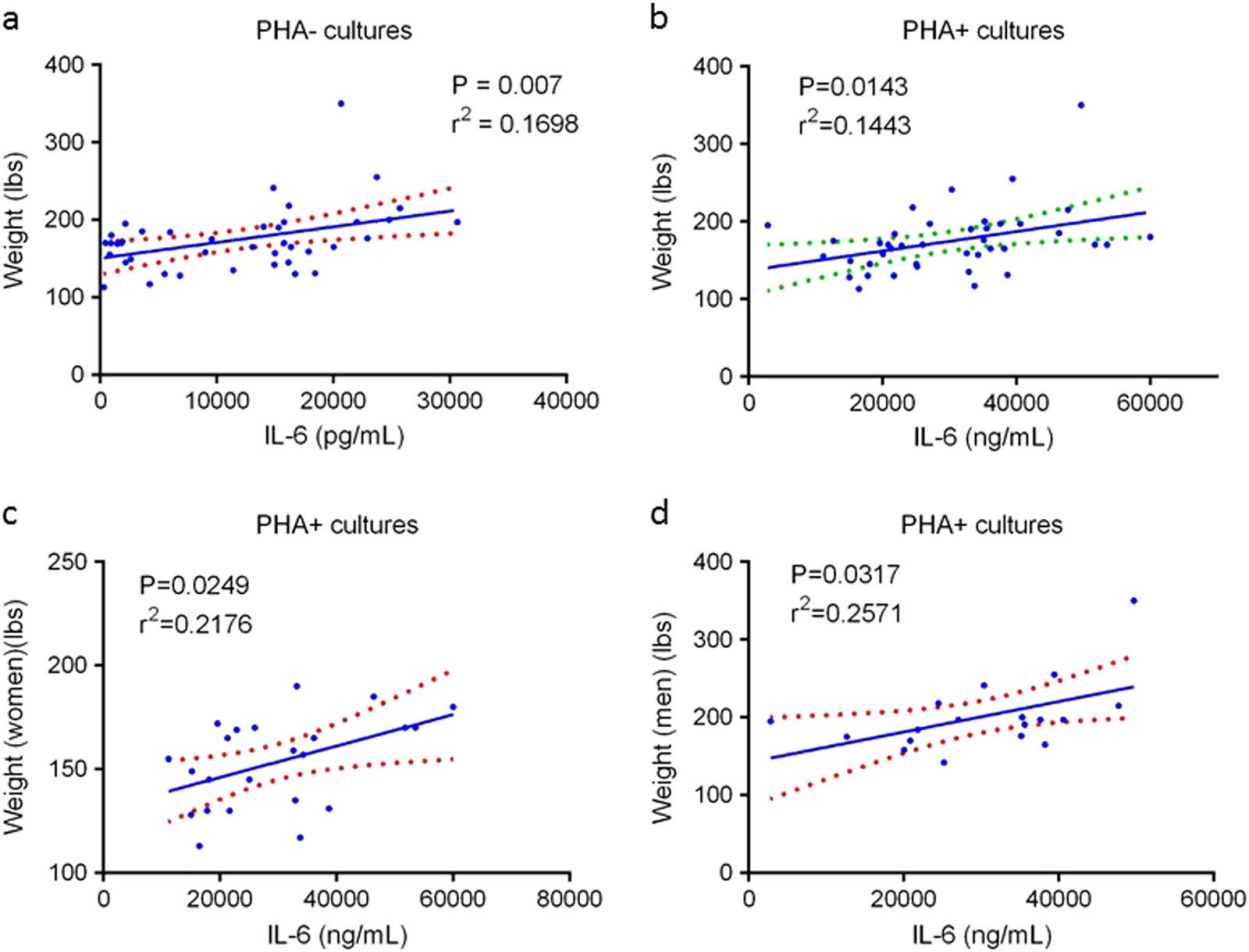
Effect of body weight on IL-6 production by cultured peripheral blood mononuclear cells. This figure is from a publication on the effect of long-term exercise on peripheral blood mononuclear cell (PBMC) production of osteoclastogenic and anti-osteoclastogenic cytokines.^4^ **a** Spontaneous production of IL-6 by cultured PBMC (N = 41 subjects). **b** IL-6 production in PBMC cultures containing the T-cell mitogen phytohemagglutinin (PHA) (*N* = 41 subjects). **c** IL-6 production by PBMC of women in PHA + cultures (*N* = 23 subjects). **d** IL-6 production by PBMC of men in PHA + cultures (*N* = 18 subjects). (Linear regression analyses with 95% confidence intervals)

In a recent study involving 43 healthy adults,^4^ we found that 6 months of combined resistance and aerobic exercise training diminished bone resorption and enhanced bone formation by changing the proportions of peripheral blood mononuclear cells (PBMC) producing osteoclastogenic cytokines (interleukin (IL)-1α, tumor necrosis factor (TNF)-α, and interferon (IFN)-γ) and those producing anti-osteoclastogenic cytokines (IL-4, IL-6, IL-10, and transforming growth factor (TFG)-β1). Notably, post-exercise increases in IL-6, a pleotropic cytokine involved in bone and muscle homeostasis and immune regulation,^5^ were proportionate to body weight, a measure of one’s mass times the intensity of the gravity field (9.8 /m/sec^2^ on Earth) (Fig. 1). This finding raises the possibility that the failure of exercise programs to attenuate bone and muscle loss in the microgravity of space is related to suboptimal production of this important cytokine by postural (antigravity) muscles and by bone cells and their supporting mesenchymal and immune cell network.

## IL-6

IL-6 is a functionally pleiotropic growth and differentiation cytokine with context-dependent inflammatory and anti-inflammatory properties; it plays an important regulatory role in innate and adaptive immunity, hematopoiesis, and in bone, muscle, and metabolic homeostasis. It is produced after stimulation by most nucleated cells, including monocytes, macrophages, endothelial cells, fibroblasts (main sources), T cells, B cells, granulocytes, mast cells, myocytes, osteoblasts, osteoclasts, osteocytes, chondrocytes, glial cells and keratinocytes. The main cellular targets are hepatocytes, leukocytes, T cells, B cells, and hematopoietic cells.^6^

Binding of IL-6 to its transmembrane receptor (mIL-6R) leads to a complex consisting of two IL-6 molecules (homodimer), two IL-6R proteins, and two glycoprotein 130 (gp130) molecules. Dimerization of gp130 activates cytokine receptor-associated Janus tyrosine kinases (primarily Jak 1); in turn, the kinases activate cytoplasmic signal transducer and activator of transcription 3 (STAT3) which dimerizes and translocates to the nucleus where it initiates transcription. The negative feedback of this signaling pathway is regulated by suppressor-of-cytokine-signaling (SOCS) proteins 1 and 3 and the protein inhibitors of activated STATs.^6,7^

Although expression of mIL-6R is restricted, being found primarily on hepatocytes and some leukocyte subsets, IL-6 also interacts with soluble IL-6R (sIL-6R), which on binding to its ubiquitously expressed gp130 co-receptor can activate a variety of cells (IL-6 trans-signaling). During inflammation, mIL-6R is cleaved by the metalloprotease ADAM 17 and shed as sIL-6R from activated cells, markedly increasing IL-6 trans-signaling and expanding the sphere of influence of this key cytokine.^6,7^ Low levels of soluble isoforms of IL-6-R and gp130 normally present in blood serve to neutralize non-inflammatory levels of IL-6, thus protecting cells from overstimulation by IL-6 *trans*-signaling.^7^

IL-6 *trans*-signaling is primarily responsible for the proinflammatory activities of IL-6, whereas signaling via mIL-6R accounts for most of its anti-inflammatory and metabolic activities.^7^

## IL-6 and immunity

### On Earth

#### Innate immunity

In the early immune response, the source of IL-6 is primarily from innate immune cells activated by toll-like receptor (TLR) binding of pathogen-associated molecular patterns (PAMPs) and by the secretion of IL-1α/β, TNF-α, IFN-γ, and/or granulocyte-macrophage colony stimulating factor (GM-CSF) by monocytes and macrophages.^6^ IL-6 usually enhances TLR-mediated cytokine and chemokine production; however, IL-1β, TNF-α, and IL-8 (CXCL8) production is suppressed by IL-6 when TLR4 binds its ligand, lipopolysaccharide (LPS), thus providing protection against endotoxemia.^8^

IL-6 plays an important role in leukocyte trafficking and the transition from neutrophilic to monocytic infiltration at sites of inflammation.^9–11^ IL-6 trans-signaling promotes leukocyte recruitment to sites of acute inflammation by upregulating endothelial cell (EC) expression of intracellular adhesion molecule-1 (ICAM-1) and chemokines.^9^ By inducing monocyte expression of macrophage colony stimulating factor receptors, IL-6 promotes the differentiation of monocytes to macrophages.^6^ It also can cause dendritic cells to differentiate into macrophages^12^ and activate anti-inflammatory IL-10^+^ M2-like (M2d) macrophages.^13^

#### Humoral immunity

IL-6 controls the proliferation, maturation and survival of B cells and plasmablasts and initiates T-cell-dependent isotype switching and antibody production. In conjunction with IL-1β as a cofactor, IL-6 prompts the differentiation of IL-10^+^ B regulatory (B1) cells^6^ and triggers IL-21 production in CD4^+^ T cells to drive STAT-3-dependent plasma cell development.^14^ IL-6 is essential for T follicular cell (Tfh) differentiation; these cells are critical to the ability of B cells to undergo isotype switching, terminal differentiation, and high affinity antibody production.^15^

#### Cell-mediated immunity

IL-6 regulates the trafficking of CD4^+^ T cells, CD8^+^ T cells, natural killer (NK) cells, dendritic cells, monocytes and macrophages by upregulating the expression of chemokines CCL4, CCL5, CCL11, CCL17 and intracellular and vascular adhesion molecules ICAM-1 and VCAM-1.^6^ It is an important growth and differentiation factor for activated naïve CD4^+^ T cells: by upregulating their expression of nuclear factor of activated T cells (NFATc2) or SOCS1, IL-6 promotes IL-4-mediated Th2 cell differentiation^16^ or inhibits IFN-γ-mediated Th1 differentiation,^17^ respectively. With TGF-β as a cofactor, IL-6 drives the differentiation of IL-21-producing Th17 cells; it can also convert naturally occurring T regulatory (Treg) cells into Th17 cells^18^ and increase Treg cell maturation by triggering the production of IL-27 by monocytes and macrophages.^19^

IL-6 is also an important growth and differentiation factor for activated naïve CD8^+^ T cells. It can trigger their differentiation into IL-21-producing cells with activity against the influenza virus^20^ and synergize with IL-15 or IL-7 to stimulate T-cell receptor (TCR) independent proliferation and effector functions.^21^ In conjunction with IL-27 as a cofactor, IL-6 prompts the secretion of IL-10 by IFN-γ^+^ Th1, IL-4^+^ Th2 and IL-17^+^ Th17 cells. It also facilitates T-cell survival, inhibits TNF-α and IL-1β synthesis and induces IL-2 secretion and IL-2R expression in T cells. With IL-3 as a costimulant, IL-6 initiates the proliferation of multipotential hematopoietic cells, including the maturation of megakaryocytes.^6^

#### Acute Phase Response

IL-6 synergizes with IL-1β, TNF-α, TGF-β, IL-8, and IL-22 to increase the production of acute phase proteins (APP) by hepatocytes.^22^ APP such as C-reactive protein play a protective role at sites of inflammation and infection.^23^

## In Space

### Innate immunity

#### Studies in humans

Studies on monocytes, macrophages, and TLRs done immediately after spaceflight have provided mixed results. Berendeeva and associates documented variable increases in the relative and absolute counts of PBMC expressing TLR2, TLR4, and TLR6,^24^ findings like those described by Ponomarev et al. who found an increase in serum levels of TLR2 and TLR4 ligands (HSP60, and HSP70 and HMG1, respectively) accompanied by an increase in the number of leukocytes expressing TLR2 and TLR4.^25^ In contrast, Rykova reported a decrease in the levels of circulating monocytes and granulocytes expressing TLR2, TLR4 and TLR6, accompanied by a reduction in TLR4-mediated LPS-induced cytokine production.^26^

Crucian and associates found that monocyte expression of cell surface markers important for antigen presentation (HLA-DR) and for adhesion and tissue migration (CD62L) as well as the production of IL-6, IL-10 and TNF-α by LPS-stimulated monocytes were reduced following short-duration spaceflight. Importantly, only IL-6 production was diminished in response to the pan-leukocyte mitogen phorbol myristate acetate (PMA) + ionomycin.^27^ In a later study involving in-flight sampling, Crucian and associates found that IL-8 secretion was elevated in LPS-stimulated monocytes but there was no statistically significant change in the production of IL-12, TNF-α, IL-10, IL-6, or IL-1-β.^28^

Compared to non-astronaut ground controls, Kaur and associates found that intracellular levels of IL-6 and IL-1β were reduced and levels of IL-8 and IL-1 receptor antagonist (IL-1ra), were increased in LPS-stimulated monocytes taken both before and after spaceflight; except for IL-1-ra values returned to normal 6–12 months post-spaceflight.^29^ They also found that blood monocytes had a reduced ability to phagocytose *Escherichia coli*, elicit an oxidative burst, and degranulate; this impairment was accompanied by a reduced expression of two surface markers involved in phagocytosis, CD32 and CD64.^30^

#### Studies in rodents and drosophila

Studies on the effects of spaceflight and simulated microgravity on murine splenocytes have also produced mixed results. Shen-An Hwang and associates found that a 13-day spaceflight increased the percent of dendritic cells expressing MHC I (CD11c^+^MHC I) and the ability of splenic macrophages to phagocytose fluorescent-tagged beads and to produce TNF-α when stimulated with a TLR-2 agonist but not when stimulated with the TLR4 agonist LPS; IL-6 production was unchanged as compared to ground controls.^31^ In contrast, Wang et al. using simulated microgravity found that LPS-induced TNF-α expression was impaired due to activation of heat shock factor-1, a known repressor of the TNF-α promoter.^32^ Also using simulated microgravity, Brungs et al. found that splenic macrophages cultured with TLR-agonists had impaired production of reactive oxygen species (ROS) caused by diminished tyrosine kinase phosphorylation.^33^ Taylor and associates found that spaceflight produced stress-related transcriptional responses that diminished the ability of Drosophila to mount a TLR-mediated response to fungal infection.^34^

### Humoral Immunity

#### Studies in humans

There are comparatively few studies on B-cell function in space. Voss found there no significant changes in serum immunoglobulin levels following a 10-day spaceflight^35^ and Mills and associates found that mean circulating levels of CD19^+^ B cells increased in 11 astronauts following five 4–16-day shuttle flights.^36^

#### Studies in rodents and amphibians

Spaceflight is reported to cause reductions in blood and splenic levels of B cells in rodents.^37,38^ In addition, studies in the amphibian *Pleurodeles waltl* have shown that spaceflight can cause impaired antibody responses and changes in IgM heavy-chain transcription.^39^

### Cell-Mediated Immunity

#### Studies in humans

Studies in astronauts have shown impaired delayed cutaneous hypersensitivity reactions and an increased incidence of herpes-group virus shedding during spaceflights.^40^ Crucian and associates found that virus-specific T-cell function was diminished in 19 astronauts during 10–15-day shuttle flights; they also noted in-flight dysregulation of CD8^+^ T-cell subsets and diminished leukocyte secretion of IFN-γ, TNF-α, IL-10, IL-4, IL-5 and IL-6 in response to PMA + ionomycin; importantly, these authors noted significant differences in samples taken in-flight as compared to those taken post-flight.^28^ Other studies have documented post-flight reductions in blood levels of CD4^+^ T cells, CD8 ^+^ T cells, and NK cells, increases in the proportion of T helper type 2 cells and CD4/CD8 ratios, decreases in the cytotoxicity of NK cells, altered plasma levels of cytokines, and variably impaired or enhanced secretion of Th-1 and Th-2-type cytokines in response to T-cell-receptor-specific and/or non-specific mitogens.^36,41–44^

#### Studies in rodents

In rodents, space travel decreases the masses of lymph nodes, thymuses, and spleens and alters the distribution of CD3^+^, CD4^+^, and CD8^+^ T cells and NK cells in blood, spleen, and/or lymph nodes.^37,38,45–47^ Also documented are consistent decreases in mitogen-induced secretion of IL-2.^38,46-49^ Spaceflight and simulated microgravity have been shown to reduce IL-2, IL-2Rα, and IFN-γ gene expression and secretion in anti-CD3/CD28 antibody-activated mouse splenocytes.^50^

## IL-6 and bone homeostasis

### On earth

IL-6 promotes bone formation by enhancing the differentiation of osteoblasts precursors and by protecting osteoblasts from apoptosis.^51–55^ It protects against bone resorption by decreasing receptor activator of nuclear factor kappa B ligand (RANKL) expression in osteoclasts, and by stimulating the production of the anti-osteoclastogenic cytokines IL-4, IL-10, and IL-1 receptor antagonist while inhibiting the production of the osteoclastogenic cytokines IL-1-α/β and TNF-α by immune cells.^56,57^ IL-6 regulates the expression of osteoprotegerin (OPG) in murine calvariae,^58^ and is the main growth factor for B cells, the chief source of OPG in bone marrow stroma;^59^ OPG is a potent inhibitor of osteoclastogenesis, and has recently been shown to reduce bone resorption when administered to mice during spaceflight.^60^

IL-6 is produced in osteocytes and osteoblasts in response to bone loading signals and plays an important role in bone remodeling.^61–63^ In murine osteoclasts, IL-6 binding to IL-6R and its coreceptor, gp130, releases osteoclast-derived coupling factors and osteotransmitters that protect bone by upregulating osteoblast activity.^64^

### In Space

Space travel has been shown to accelerate astronaut bone loss to 1–1.6% per month, primarily in weight bearing bones^65^; this loss is associated with an increase in bone resorption and a decrease in bone formation^66^ and has been attributed to the reduction in bone-loading signals normally transduced by osteocytes resident in the lacunar-canalicular network of bone.^67^ Researchers have found that osteocyte apoptosis in trabecular and cortical bone occurs within 3 days of simulated weightlessness in mice and precedes recruitment of osteoclasts.^68^ In addition, modeled microgravity and hindlimb unloading has been shown to induce osteoclast precursors to enhance RANKL-mediated osteoclastogenesis.^69^

Bone formation is reduced, and bone remodeling is impaired in IL-6 knockout (IL-6^-^/^-^) mice,^70^ and Harris and associates found that orbital spaceflight caused impaired osteoblast function associated with a reduction IL-6 mRNA expression.^71^ Using cultures of murine osteoblasts, osteocytes, osteoclast precursors, and compressive cyclic forces, Hao and associates found that IL-6 + sIL-6R increased osteocyte-mediated osteoblast differentiation and inhibited osteoclastogenesis and osteoclast differentiation under mechanical loading via STAT3 and extracellular signal-regulated kinase (ERK) signaling pathways^72^

## IL-6 and muscle homeostasis

### On Earth

IL-6, a myokine, plays an important role in energy homeostasis and repair and remodeling of skeletal muscle. It activates skeletal muscle 5‘ adenosine monophosphate activated protein kinase (AMPK) and/or phosphatidylinositol-3-kinase (P13K), increasing glucose uptake and mitochondrial oxidation of fatty acids and enhancing exercise endurance.^73,74^

IL-6 also plays a “pivotal” role in the response of skeletal muscle to injury which is determined by both existing muscle fiber nuclei (which are terminally differentiated) and by a population of multi-potential Pax7^+^mononucleated satellites cells (SC).^75^ When activated, SC migrate to the site of injury or remodeling and, under the control of a network of transcription factors, proliferate and differentiate into myocytes.^75,76^ IL-6 MKO mice have reduced levels of SC proliferation and muscle repair capacity,^77^ and Ring finger protein-13 (RNF-13) knockout mice have accelerated skeletal muscle regeneration mediated in part by macrophage-secreted IL-6.^78^ IL-6 upregulates the secretion of IL-4 and IL-10 in immune cells and both cytokines play a positive role in myogenesis.^75,78^ By increasing IL-6 secretion by myocytes, exercise has been shown to promote extracellular matrix reorganization and stem cell accumulation in the skeletal muscle stem cell niche.^79^

### In Space

In both humans and rodents, the primary effect of spaceflight on skeletal muscle is fiber atrophy resulting in a decline in peak force, power, and exercise tolerance^80^; the atrophy mainly involves antigravity muscles such as the soleus.^81^ At a molecular level, microgravity-induced atrophy is due to increased proteasome activity coupled with a reduction in protein synthesis and mitochondrial biogenesis.^82^ As noted above, IL-6 plays an important role in muscle repair and myogenesis.

Skeletal muscle-specific AMPKα1α2 knockout mice (mdKO) have reduced exercise performance and fatigue resistance,^83^ findings similar to those described in humans and rodents during space flight. IL-6 knockout mice (IL-6 KO) and muscle-specific IL-6 knockout mice (IL-6 MKO) have similar decreases in exercise tolerance,^84^ presumably due to a reduction in IL-6-mediated AMPK secretion by exercising muscle. The observation that weightlessness impairs the ability of murine soleus muscles to oxidize free fatty acids^81^ suggests that IL-6-mediated AMPK activation is impaired in weight bearing muscles during spaceflight. Normally during prolonged exercise there is a shift from carbohydrate utilization to lipid oxidation, thereby enhancing exercise tolerance.^84^

In a study done on mice during a 91-day spaceflight, Sandona and associates found that soleus muscles lost ~35% of cross-sectional area whereas extensor digitorum longus (EDL) muscles showed no atrophy.^81^ Soleus muscles underwent physiological and morphological transformations, changing to a faster, more glycolytic phenotype, with reductions in the proportion of slow twitch type 1 and 2 A fibers, increases in the proportion of fast twitch 2X and 2B fibers, and corresponding changes in myosin heavy-chain isoforms. Gene expression of muscle-specific growth factors IL-6 and insulin-like growth factor (IGF)-1 was downregulated in soleus muscles and upregulated in EDL muscles. EDL muscles also upregulated gene expression of stress-related markers. They concluded that, in contrast to soleus muscles, EDL muscles compensate for the effects of microgravity by increasing the expression of IL-6 and IGF-1 and various stress proteins; they posited that IGF-1 and IL-6 may be good candidates to counter the adverse effects of space travel on antigravity muscles such as the soleus.

## IL-6 and metabolic homeostasis

### On Earth

#### Central nervous system

When injected into the ventricles of obese rats, IL-6 has been shown to restore the anorexigenic effects of insulin and leptin by promoting IL-10-mediated inhibition of I_K_B kinase β/NFκB signaling and endoplasmic reticulum stress responses.^85^ IL-6 knockout mice develop late-onset obesity and glucose intolerance.^86^

#### Pancreas

Using wild-type and IL-6 knockout mice with type 1 diabetes, Paula and associates found that exercise-induced generation of IL-6 increased β-cell viability in cultured pancreatic tissue by reducing the proinflammatory effects of IL-1β and IFN-γ.^87^

IL-6 has also been shown to increase insulin secretion by promoting the production of the anorexigenic incretin glucagon-like peptide-1 (GLP-1) by intestinal L and alpha cells.^88^

#### Liver

In diet-induced obese rodents, IL-6 increases mitochondrial β-oxidation of fatty acids in hepatocytes, alleviating hepatic steatosis.^89^

#### Adipocytes

Approximately one-third of IL-6 is estimated to originate from adipose tissue where its effects are largely anti-obesogenic and anti-inflammatory. IL-6 stimulates lipolysis and fat oxidation in adipocytes,^90^ downregulates TLR4-induced TNF-α, IL-8, and macrophage metalloproteinase-1 (MCP-1) production by resident macrophages, and prevents mature onset obesity and insulin resistance in mice.^91^

#### T cells

Activated T cells undergo metabolic reprogramming that promotes glycolytic flux and lactate production and increases the production of lipids, proteins, nucleic acids and other carbohydrates. Mammalian target of rapamycin (mTOR) signaling promotes Th1, Th2, and Th17 differentiation, whereas Treg cells are generated when AMPK signaling is activated and mTOR activation is suppressed. Unlike effector CD4^+^ and CD8^+^ T cells, Tregs and memory T cells oxidize fatty acids for fuel. Upon activation, T cells also express insulin and leptin receptors and become sensitive to insulin signaling and nutrient availability.^92^ Accumulation of lactate and lactic acid at sites of inflammation has been shown to differentially inhibit the motility of CD4^+^ T cells and CD8^+^ T cells by their effects on subtype-specific transporters Sic5a12 and Sic16a1, respectively.^93^

As previously noted, IL-6 stimulates AMPK activity in myocytes; it is unclear as to whether this also occurs in T cells.

### In Space

#### Pancreas

Subclinical diabetogenic changes, including alterations in insulin secretion, insulin sensitivity, glucose tolerance, and metabolism of protein and amino acids occur during spaceflight and in simulated conditions of microgravity. Experiments in flight and after flight, ground-based bedrest studies, and bioreactor studies of pancreatic islets of Langerhans indicate that the pancreas is unable to overcome peripheral insulin resistance and amino acid dysregulation that occurs during space flight.^94^

#### Liver

Pecaut and associates measured liver transcriptomics and metabolomics in female C57BL/6J mice after a 13-day flight on the space shuttle Atlantis. Although the livers were depleted of glycogen, functional gene analysis revealed both an increase in glycogen synthesis and glycogenolysis, pathways that do not normally occur simultaneously except in the glycogen-depleted liver. They also noted an increase in hepatic fatty acid oxidation.^95^

#### Adipocytes

Spaceflight is associated with bone marrow adipogenesis due to redirected morphogenesis of mesenchymal cells.^96^ However, there is little in the literature documenting metabolic changes in adipocytes during spaceflight.

### T cells

Using in-flight experiments and blood from human donors, Chang and coworkers have shown that Con A and anti-CD28-stimulated T-cell activation is impaired in microgravity due to down-regulation of Rel/NF-κB, CREB, and SRF gene targets. The TNF pathway was the major early downstream effector pathway inhibited, potentially contributing to ineffective proinflammatory responses during spaceflight.^97^

### Recombinant IL-6: effect and safety issues in humans

The reader is referred to a comprehensive review by Kammüller on safety issues raised by the use of recombinant human IL-6 (hrIL-6) as a therapeutic agent.^98^ Provided below are several studies on the immune and metabolic effects of rhIL-6 in humans using well tolerated doses that achieve plasma levels similar to those reached during strenuous exercise.

Steensberg and associates administered recombinant human IL-6 (rhIL-6) intravenously at a rate of 30 µg/hour for three hours to six healthy young men achieving plasma levels of ~140 pg/mL (equivalent to levels obtained during strenuous exercise) which declined to preinfusion levels within an hour post-infusion. rhIL-6 was well tolerated, with no changes noted in temperature, heart rate, blood pressure, or plasma epinephrine levels. Plasma levels of the anti-inflammatory cytokines IL-10 and IL-1ra increased significantly during the infusions (8-fold and 26-fold, respectively) whereas there were no changes in plasma TNF- α levels; plasma cortisol levels also increased causing a transient neutrophilia and lymphopenia. CRP levels rose 3 and 16 h post-infusion.^99^

In a study involving 18 healthy men receiving intravenous glycerol and palmitate, Van Hall and associates found that both low and high dose rhIL-6 stimulated lipolysis and fat oxidation. Those receiving low dose rhIL-6 had mean plasma levels of 140 pg/mL and experienced no adverse side effects, whereas those receiving high dose rhIL-6 had mean plasma levels of 319 pg/mL and developed ~30 min of “chills and discomfort”. Plasma levels of insulin and glucagon were unaffected, whereas plasma adrenalin levels increased in the high dose group. Cortisol levels rose in both treatment groups, retuning to base line within 2 h post-infusion.^100^ In a study involving eight healthy men the same group found that a 4-h infusion of low dose rhIL-6 (30 µg/h) selectively stimulated lipolysis in skeletal muscle but not in adipose tissue. Again, this dose of rhIL-6 was well tolerated.^101^

The reader is referred to Table 1 for a summary of the pleiotropic effects of IL-6.

**Table 1:** Pleiotropic effects of IL-6

TARGET	EFFECT
B cells	Controls the proliferation, maturation and survival of B cells and plasmablasts; initiates T-cell-dependent and -independent isotype switching and antibody production; promotes the differentiation of IL-10^+^ B regulatory (B1) cells [IL-1β], and IL-21 production in CD4^+^ T cells to drive STAT-3 dependent plasma cell development.
T cells	Regulates trafficking of lymphocytes, monocytes and macrophages and initiates transition from granulocytic to mononuclear cell infiltration at sites of inflammation; upregulates expression of surface markers involved in antigen presentation and phagocytosis; promotes the differentiation of Th2 [IL-4], Th17 [TGF-β], Th22 [TNF-α], Treg [IL-27], and Tfh [IL-21] cells; initiates the secretion of IL-10 by IFN-γ^+^ Th1, IL-4^+^ Th2 and IL-17^+^ Th17 cells [IL-27]; facilitates T-cell survival; inhibits Th1 differentiation [IFN-γ] and TNF-α and IL-1β secretion; enhances IL-2, IL-4, IL-10, IL-1ra secretion and IL-2R expression; stimulates TCR independent CD8^+^ T-cell proliferation and effector functions [IL-7 or IL-15].
Monocytes, dendrocytes	Promotes monocyte and dendrocyte to macrophage differentiation and IL-10^+^ M2 macrophage (M2d) activation.
Hematopoietic progenitors	Promotes proliferation of multipotential hematopoietic cells, including the maturation of megakaryocytes [IL-3].
Hepatocytes	Initiates acute phase protein synthesis.
Bone	Promotes bone formation by enhancing OB differentiation from mesenchymal cell precursors, by inhibiting OB apoptosis, and by augmenting immune cell secretion of IL-4, IL-10 and IL-1ra; inhibits bone resorption by decreasing OC RANKL expression, by upregulating OPG secretion in bone and B cells, and by inhibiting immune cell secretion of IL-1α/β and TNF-α; enhances OB activity in response to bone loading signals by releasing osteoclast-derived coupling factors/transmitters.
Muscle	Increases glucose uptake and mitochondrial fatty acid oxidation by activating AMPK, P13K. Promotes myocyte differentiation, proliferation, and response to injury. Promotes post-exercise extracellular matrix reorganization and stem cell niche accumulation.
Metabolic homeostasis	Restores CNS sensitivity to insulin, leptin; prevents obesity, glucose intolerance; increases pancreatic beta cell viability and insulin secretion; induces lipolysis, fat oxidation in hepatocytes, adipocytes.

Cytokines bracketed by [] act as essential cofactors

*AMPK* 5′ adenosine monophosphate activated protein kinase, *CNS* central nervous system, *G/M* granulocyte/monocyte, *IFN-γ* interferon-γ, *IL* interleukin, *IL-1ra* interleukin-1 receptor antagonist, *IL-2R* interleukin 2 receptor, *OB* osteoblast, *OC* osteoclast, *OPG* osteoprotegerin, *P13K* phosphatidylinositol-3-kinase, *RANKL* receptor activator of nuclear factor kappa B ligand, *TCR* T-cell receptor, *Th* T helper, *Tfh* T follicular helper, *TGF-β* transforming growth factor-β, *TNF-α* tumor necrosis factor-α

## Discussion

During spaceflight astronauts and cosmonauts experience a unique set of stressors including the effects of microgravity, suboptimal nutrition, social isolation, confinement, sleep deprivation, deconditioning, atypical work environment, solar radiation, and alterations in circadian rhythms.^28,40,102^ Also extant are pre- and postflight stressors, most notably those associated with landing and the abrupt need to re-adapt to Earth’s gravity.^28^ And, by necessity, studies on the immune system and bone and muscle homeostasis have involved different flight times and variations in research protocols. In this regard, most space physiologists identify the results of studies performed during long duration orbital spaceflights as being much more analogous to future deep space missions than short duration shuttle missions.

Despite these difficulties, studies have consistently shown that spaceflight is associated with immune dysregulation, including alterations in surface markers, tissue distribution, cytokine production, phagocytic capacity, and anti-viral activity of immune cells,^24–50^ Also well documented are the accelerated losses in bone and muscle mass and the loss of muscle strength and endurance during spaceflight.^65–72,80–84^

We have previously reported that long-term moderate intensity exercise increases the proportion of PBMC producing anti-inflammatory cytokines and cytokines with osteogenic and myogenic properties, and that these changes are associated with reductions in serum markers of bone resorption, increases in markers of bone formation, and improvements in exercise tolerance and muscle strength.^4^ Because PBMC constantly circulate through the highly vascular networks of bone and muscle, they have the potential, supported by our findings, to influence the physiology and ontogeny of muscle cells, and osteoclasts, osteoblasts, osteocytes, and their precursors. PBMC preparations also contain multipotential stem cells capable of differentiating into a variety of tissues, including myocytes and bone cells.^103^

In our study, IL-6 was the only cytokine whose secretion was proportionate to body weight, a measurement of the force of gravity. In this regard, Wehland and associates, found that IL-6 secretion in human chondrocytes increased > 2-fold when the cells were cultured under conditions of hypergravity (1.8 g),^104^ and Ma and associates documented a significant increase in IL-6 gene activation in thyroid cancer cells cultured under similar levels of hypergravity.^105^ In contrast, IL-6 production by mitogen-stimulated human PBMC is reduced in the microgravity of long duration spaceflight,^106^ and short-duration space flight has been found to dysregulate monocyte phenotype and reduce LPS-stimulated monocyte expression of several cytokines, including IL-6; notably, in this study, only IL-6 secretion was reduced postflight in blood leukocytes cultured in the presence of phorbol myristate and ionomycin.^27^ And, as previously noted, IL-6 gene expression in murine soleus muscle has been shown to decrease in the microgravity of spaceflight,^81^ and spaceflight impairment in osteoblast function has been attributed to a reduction IL-6 mRNA expression.^71^

As documented in this review, reductions in the secretion of IL-6 during spaceflight could adversely affect a variety of its immune functions. This includes: the expression of intracellular and surface markers involved in leukocyte trafficking and the transition of neutrophilic to monophilic inflammatory responses; the expression of surface markers involved in antigen presentation and phagocytosis; the augmentation of IL-2, IL-2R, IL-4, IL-10 and IL-1ra and inhibition of IL-1β and TNF-α production; the promotion of T and B-cell survival; the enhancement of T-cell-dependent and -independent antibody production; the differentiation of monocytes, macrophages, dendritic cells, B cells, and plasmablasts; the initiation of the acute phase protein response and the proliferation of multipotential hematopoietic cells, including the maturation of megakaryocytes, and, as a cofactor, the differentiation of CD4^+^ and CD8^+^ T cells, Th1 and Th2 cells, Th17 cells, Tregs, Tfh cells.^6,9–22,27^

Reductions in IL-6 secretion could also contribute to the development of subclinical diabetes (pre-diabetes) reported to occur during spaceflight since, as previously noted, IL-6 can increase pancreatic beta cell viability, insulin secretion, and CNS insulin sensitivity, and prevent the occurrence of obesity, glucose intolerance, and insulin resistance in mice fed obesogenic diets.^85–88,94^

D-P Häder and associates in reviewing gravireceptors in eukaryotes noted that many eukaryotes use a mass such as a statolith or total cell content to operate on gravireceptors (in many cases a mechanosensitive ion channel) either by pulling or pressing on an element of the cytoskeleton, ultimately resulting in the activation or silencing of genes. In human cells, they noted a direct correlation between changes in the cytoskeleton and transcription alterations in microgravity and posit that the adhesive interaction of the cytoskeleton with the extracellar matrix is the basis for gravisensing.^107^ Whatever the mechanism, microgravity alters the expression of several transcription factors, including nuclear factor kappa-light-chain-enhancer of B cells (NF-kB); NF-kB upregulates IL-6 production in human lung epithelial cells and smooth muscle cells when their cytoskeletons are subjected to mechanical stress or stretching.^108,109^ And cardiac muscle AMPK expression is downregulated in microgravity^110^; as noted previously, IL- 6 enhances exercise endurance by activating AMPK-mediated increases in glucose uptake and fat oxidation within myocytes.^73,74^

## Conclusion

It is posited that secretion of IL-6 is particularly sensitive to cytoskeletal derangements and extracellular adhesive changes that occur under the force of gravity on Earth and in the microgravity of spaceflight, and that the adverse effect of spaceflight on immune, bone, muscle and metabolic homeostasis is related, at least in part, to altered gravisensing and consequent suboptimal production of this key cytokine.

### Future direction

On Earth, plasma levels of IL-6 increase in an exponential fashion (up to 100-fold) in response to exercise and decline rapidly in the post-exercise period. The increase is related to exercise intensity, duration, the mass of muscle recruited and one’s endurance capacity.^57^ Preflight and inflight studies measuring post-exercise IL-6 plasma levels should be done to determine whether production of this key myokine/cytokine is reduced during spaceflight, in which case inflight administration of rhIL-6 may prove useful in preventing some of the deleterious effects of spaceflight, particularly on muscle and bone.

## Data Availability

The data used in figure one are available from the author on request.
